# Isolation, Diversity, and Growth-Promoting Activities of Endophytic Bacteria From Tea Cultivars of Zijuan and Yunkang-10

**DOI:** 10.3389/fmicb.2018.01848

**Published:** 2018-08-21

**Authors:** Xiaomei Yan, Zhi Wang, Yu Mei, Liqun Wang, Xu Wang, Qingshan Xu, Su Peng, Yu Zhou, Chaoling Wei

**Affiliations:** State Key Laboratory of Tea Plant Biology and Utilization, Anhui Agricultural University, Hefei, China

**Keywords:** endophytic bacteria, Zijuan, Yunkang-10, *Camellia sinensis* var. *assamic*, plant growth promoting

## Abstract

Endophytes are rich in plant tissues and play important roles in plant-microbial interactions and plant-growth regulation. Here, endophytic bacteria from two closely related tea cultivars of Zijuan and Yunkang-10 were isolated, and the diversities were compared. Plant-growth promoting (PGP) activities were determined on the dominant groups or nitrogen-fixing genera from the two cultivars. Endophytic bacteria were isolated by using of different selective media and methods, and the PGP activities were investigated by analytical and molecular technologies. A total of 110 isolates of 18 genera belonging to three phylums (*Proteobacteria, Firmicutes*, and *Bacteroidetes*) were obtained from Zijuan, while 164 isolates of 22 genera belonging to two phylums (*Proteobacteria* and *Firmicutes*) were obtained from Yunkang-10. PGP screening indicated that *Herbaspirillum* spp., *Methylobacterium* spp., and *Brevundimonas* spp. showed different PGP abilities. The PGP ability decreased in order of *Herbaspirillum* spp., *Brevundimonas* spp. and *Methylobacterium* spp., and the majority of *Methylobacterium* spp. did not showed PGP activity of nitrogen-fixation, P-solubilization, siderophore, indole-3-acetic acid (IAA) production or 1-aminocyclopropane-1-carboxylate (ACC) deaminase. The study of bacterial community and PGP activities confirmed that endophytes in tea plants are constantly changing in different seasons and tea cultivars, and the PGP bacteria in Zijuan are much abundant than those of Yunkang-10.

## Introduction

Endophytes are a group of microorganisms that reside in healthy plants, but not result in plant disease, and endophytes have attracted increasing attention to microbiologist (Qin et al., 2011). Numerous studies indicated that endophytes play important roles in plant disease resistance, secondary metabolites synthesis, plant growth regulations, and environmental stressors withstand (Coombs and Franco, 2003; Qin et al., 2009, 2011, 2015, 2017; Glick, 2014; Mora et al., 2014; Zhao et al., 2016). Early endophytes studies mainly focused on rice, wheat, cotton, and other staple crops, but endophytes from other plants (including tea and medicinal plants) were rarely considered (Coombs and Franco, 2003; Ji et al., 2014; Hardoim et al., 2015). Recently, endophytes have become a research hotspot in microbiology field for the abundant secondary metabolites, PGP activities and plant protection roles (Qin et al., 2009, 2015, 2017; Glick, 2014; Mora et al., 2014; Zhao et al., 2016). Hitherto, the interaction patterns between many host plants and endophytes remains unknown (Strobel and Daisy, 2003; Qin et al., 2017). Plenty of studies showed that endophytes can regulate plant growth by means of nitrogen-fixation, phosphate solubilization, siderophore, 1-aminocyclopropane-1-carboxylate (ACC) deaminase activity and indole-3-acetic acid (IAA) synthesis. The study of Zgadzaj et al. (2015) showed that endophytes and symbiotic nitrogen-fixing bacteria in nodules of leguminous plants jointly improved pathogen resistance ability of host plants. Similarly, the endophytic *Klebsiella* sp. and *Enterobacter* sp. in sugarcane significantly promoted the host plant growth by nitrogen-fixation (Zhu et al., 2012; Lin et al., 2015), and the nitrogen-fixation activity of *Klebsiella* sp. assisted the host plant growing on barren dune stubbornly (Chen et al., 2013). *Jatropha curcas* L., a fuel plant prefers to grow on barren sandy soil, contains a rich population of growth-promoting bacteria, and the nitrogen-fixing *Methylobacterium* sp. (L2-4) significantly increased the host plant growth and seed yield (Madhaiyan et al., 2013, 2014, 2015). Endophytic isolates from rice plants (*Oryza sativa*) were the earliest investigated plant endophytes, and the endophytes showed versatile PGP activities of nitrogen-fixation, phosphate solubilization, siderophore and IAA synthesis (Ji et al., 2014). Qin et al. (2014) assessed the PGP functions (i.e., ACC deaminase, IAA synthesis and nitrogen fixation) of 126 endophytic isolates from the medicinal plant *Limonium sinense* (Girard) Kuntze, and found many isolates showed PGP activities, but no further study was made on the PGP mechanisms.

Tea plants (i.e., *Camellia sinensis* var. *assamic* and *C. sinensis*) are evergreen shrub, rich in polyphenols (e.g., flavonoids and catechins), anthocyanins and theanine, and considered as a healthy drink in China and other Asian countries. As we known, catechins and theanine are considered as the most important secondary metabolites in tea plants for their healthy activities of antioxidant, antimicrobial, anticarcinogenic, anti-inflammatory, and antiarteriosclerotic properties (Ito et al., 1992; Wheeler and Wheeler, 2004; Cooper et al., 2005). Anthocyanins are the secondary metabolites extensively distribute in plants that serve as plant pigments for signaling between plants and insects (or microbes); indicating nutrient availability and antioxidant activity; coloring of fruits, vegetables, flowers, and grains; providing defense as feeding deterrents and antimicrobial agents; modulating auxin synthesis and transport (Jia et al., 2015). However, only a few studies have been carried out on tea plant endophytes, and these few studies mainly focused on endophytic fungi and their plant diseases resistance. For example, endophytic fungus *Colletotrichum gloeosporioides* isolated from healthy tea plant tissues showed a strong inhibitory activity on tea plant pathogens of *Pestalotiopsis theae* and *Colletotrichum camelliae*, and the inhibitory mechanism may attribute to the fungus’ high efficient chitinase and protease (Rabha et al., 2014). Compared to fungi, endophytic bacteria received less attention to tea plants, and the interaction studies between endophytic bacteria and host tea plant have not yet investigated. Based on the research progress of other plants, the study of diazotrophs and other PGP endophytes on non-legume plant is of great significance on plant growth regulations and agriculture sustainable development. In this study, we aim to isolate and characterize endophytic bacteria from two closest cultivars of Zijuan and Yunkang-10 at different seasons, the PGP activities of nitrogen-fixation, P-solubilization, siderophore, IAA synthesis and ACC deaminase are screened and the PGP mechanisms of nitrogen-fixation and IAA synthesis will further evaluate for the predominant groups.

## Materials and Methods

### Sample Planting and Collection

Tea cultivars of Zijuan and Yunkang-10 (*C. sinensis* var. *assamic*) origin from Yunnan Province of China were transplanted at the same nursery in Shucheng County of Anhui Province (China), and grown under natural environment with the same level of cultivation and management. In this study, 3-year-old clone cuttings of Zijuan and Yunkang-10 from Dechang tea plantation (Shucheng, China) were used as the isolation materials. The samples were obtained on April 15 (spring), August 5 (summer), October 20 (autumn), and December 20 (winter) in 2015. At the time of sampling, 15 branches (one branch from each tree) with the similar growth and no pest injury were selected, and the branches were placed in fully soaked floral foam and transported to the laboratory within 2 h for bacteria isolation.

### Determination of Catechine and Anthocyanin in Tea Plant Leaf

Extractions of catechines and anthocyanins from the leaf of Zijuan and Yunkang-10 were performed as previous at room temperature under dark condition (Jiang L. et al., 2013; Jiang X. et al., 2013). Ultra-high performance liquid chromatography (UPLC, Waters ACQUITY) with TUV detector was used for catechines and anthocyanins determination. The chromatographic separation was performed on a phenomenex Kinetex-C_18_ column (2.6 μm, 4.6 mm × 100 mm). The mobile phase A: H_2_O/HCOOH (99.5: 0.5, v/v) and mobile phase B: C_2_H_3_N: 100% were used. The phase B elution gradient was from 10 to 15% in 3 min, 15 to 20% in 2 min, 20 to 25% in 5 min, 25 to 90% in 5 min, and 90% down to 10% in 5 min for anthocyanins determination. The flow rate was 0.4 mL/min, detection wavelength was 520 nm and the injection volume was 2 μL. The phase B elution gradient increased from 5 to 7% in 4 min, remained 7% with 1 min, increased from 7 to 17% in 27 min, 17 to 90% in 8 min, and 90% to 5% in 5 min for catechins determination. The flow rate was 0.8 mL/min, detection wavelength was 280 nm and injection volume was 2 μL. The total catechins in this study are (+)-catechin, (+)-gallocatechin, (-)-epicatechin, (-)-epigallocatechin, (-)-epicatechin-3-gallate and (-)-epigalloccatechin-3-gallate, and the total anthocyanins are scabiolide, delphinidin and pelargonin.

### Isolation Procedures and Cultivate Media

Leaf samples were surface-sterilized by the method as described by Qin et al. (2009) with minor modification. The samples were sterilized in the following order: a 1-min soaking in 70% ethanol, followed by a 6-min soaking in 3.25% NaOCl, a 1-min soaking in 70% ethanol, and a 1-min rinse by sterile water for five times. The sterilized tissues were imprinted onto nutrient agar (NA, Difico) and tryptic soy agar (TSA, Difico), then incubated at 30°C for 1 week to ensure the sterilization effectiveness. After surface-sterilization and drying under aseptic conditions, 5-g surface-sterilized samples from three sampling branches were cut up in a sterile mortar and grinding to homogenate, followed by dilution to 10^-1^, 10^-2^, and 10^-3^ with sterile water. An aliquot of 200-μL dilutions were spread over the surface of solid media and incubated at 30°C for 5 days. Three isolation media: NA (Difico), TSA (Difico) and tea-extract agar (Basal content: tryptone 10 g, yeast extract 5 g, NaCl 5 g, agar 18 g, 1 L distilled water, pH 7.3; tea extract: 5.0 mL) were selected for the isolation. The above mentioned NA, TSA and basal content of tea-extract medium were sterilized at 121°C for 15 min. Tea extract was prepared by leaf homogenate and sterilized by 0.22 μm filter, and tea-extract medium was prepared by adding sterilized tea extract to the sterilized and slightly cooled basal medium (∼50°C). After incubation, the bacterial colonies were picked and repeatedly re-streaked onto agar plate until their purity was confirmed for 16S rRNA gene analysis.

### DNA Extraction, PCR Amplification, 16S rRNA Gene Analysis

Genomic DNA was extracted by ChargeSwitch^®^ gDNA Mini Bacteria Kit (Invitrogen, Shanghai) following the manufacturer’s instruction. The universal primers of 16SF- (AGAGTTTGATCCTGGCTCAG) and 16SR- (GGTTACCTTGTTACGACTT) were used for PCR amplification (Stackebrandt and Goodfellow, 1991). The PCR amplification was performed as described by Qin et al. (2009), and the 16S rRNA gene was sequenced by Life Technologies Inc. (Shanghai, China). The 16S rRNA gene was manually aligned with reference sequences retrieved from GenBank database^1^ and EzBioCloud (Yoon et al., 2017) following BLAST searches for fast identification. Phylogenetic tree was constructed by the software package MEGA 6.0 (Tamura et al., 2013) after multiple alignment of sequences data by CLUSTAL_X (Thompson et al., 1997). The corrected evolutionary distance was evaluated according to Kimura’s two-parameter model (Kimura, 1980), and the clustering was based on the neighbor-joining method (NJ tree, Saitou and Nei, 1987). Bootstrap analysis was applied to the tree topology by performing 1000 resamplings (Felsenstein, 1985).

### Endophytic Bacteria Diversity Analysis

The diversity index (*H′*), the evenness index (*J*), the dominance index (*D*) and the richness index (*E*) of the endophytic isolates from Zijuan and Yunkang-10 in each season were calculated and compared according to literatures (Shannon, 1948; Simpson, 1949; Menhinick, 1964; Pielou, 1969).

Diversity analysis (Shannon-Wiener index) was calculated as: $H^{\prime }\;=\;-\sum_{i=1}^{n}PiLnPi$, the uniformity analysis (evenness index) was calculated as: $J\;=\;-\sum_{i=1}^{n}PiLnPi/LnS$, the dominance index was calculated as: $D\;=\;1-\sum_{i=1}^{n}Pi^{2}$ and the richness index was calculated as: *E* = *S* - 1/*LnN*.

The *Pi* = *ni/N, ni* is the number of the species *i* in the sample and *N* is the number of total isolates in the sample, and *S* is the number of species in the sample.

### PGP Activities Assay

The dominant endophytic bacterial groups from tea cultivars of Zijuan and Yunkang-10, and the recognized PGP bacterial genera obtained in this study were selected for PGP screening and further investigation.

#### Nitrogen-Fixation Screening and Nitrogenase Activity Determination

The fresh colony was seeded onto the nitrogen-free Ashby’s mannitol agar (Sigma-Aldrich) and Nfb agar (Hopebiol, Qingdao, China), respectively. The original isolation medium of NA, TSA or tea-extract agar was set as control, the seeded plates were incubated in 30°C, and the bacterial growth was observed. The isolate displayed growth in Ashby’s mannitol agar and made Nfb agar from green to blue was considered as positive strain, positive strain was picked to the second generation test, and the positive isolate from the third generation was recognized as nitrogen-fixing bacteria. Nitrogenase is the most important enzyme involved in nitrogen-fixation and the enzyme contains of two components, commonly called molybdenum ferritin and ferritin, the genes encoding the molybdenum ferritin are *nifD* and *nifK*, and the gene encoding the ferritin is *nifH* (Li et al., 2016; Allen et al., 2017). The *nifH* is a highly conserved 360 base-pairs region which is usually considered as the molecular marker for fast determination of nitrogenase (Allen et al., 2017). The nitrogenase activity was determined by the method of acetylene reduction as described by You et al. (2005) with minor modifications. An aliquot of 200 μL fresh culture was inoculated to 20 mL of nutrient broth and incubated overnight at 30°C. Bacterial growth was collected by centrifugation and was washed twice using sterile water, and resuspended by liquid limited nitrogen culture medium (OD_600_ = 0.2). The 3 mL suspension was transferred to a 25 mL sterilized serum vial and 2.4 mL acetylene gas (99.9999%) was driven into the serum bottle, and then incubated at 30°C for 12 h. The ethylene content and the protein of bacterial suspension were determined as You et al. (2005). DNA from nitrogenase positive isolate was used for *nifH* gene amplification, the primer set and PCR conditions were followed as Poly et al. (2001). The PCR reagents were purchased from TaKaRa (Dalian, China), and the PCR product purification, DNA sequence and phylogenetic analysis were performed as the 16S rRNA gene.

### Phytohormone Indole-3-Acetic Acid (IAA) Production Assay

Indole-3-acetic acid production was examined for the dominant endophytic groups of the tea cultivars using the method as Glickmann and Dessaux (1995). The isolate was grown in test tube containing 3 mL of dextrose-minimal salts broth [containing 1.0% Dextrose, 0.1% (NH_4_)_2_SO_4_, 0.2% K_2_HPO_4_, 0.05% MgSO_4_⋅7H_2_O, 0.01% NaCl, 0.05% CaCO_3_, 0.05% Yeast extract, PH 7.2] supplemented with 0.5 mg/mL tryptophan. The strain was incubated for 2 days at 28°C with 150 rpm, and IAA was determined by spectrophotometry as described by Qin et al. (2014). The positive isolates in preliminary screening were randomly selected for further determination of IAA synthesis dynamics. After incubation, IAA was extracted and determined by UPLC as described by Tien et al. (1979). Chromatographic separation was performed on a reverse-phase column (ACQUITY UPLC^®^ BEH C_18_ 1.7 μm 2.1 × 100 mm) in a Waters Associates liquid chromatograph (ACQUITY UPLC H-Class) equipped with an ultraviolet detector at 280 nm. The injection volume is 5 μL and the mobile phase was acetonitrile: water: acetic acid (85:15:1, v/v/v) at the flow rate of 0.5 mL/min. An Agilent 6500 UPLC-MS system (Q-TOF) was used for LC-MS qualitative analyses. The UPLC-MS separation mobile phase and the chromatographic column were the same as described for UPLC analysis. The flow rate was 0.2 mL/min and the injection volume was 2 μL. The optimum mass spectra, collision energy values and fragmentor energy were obtained by infusion of 20 μg/mL IAA standard directly in positive ionization mode. The ESI conditions were as follow: gas temperature 300°C, gas flow rate 8.0 L/min, sheath gas temperature 300°C, sheath gas flow rate 11.0 L/min, and nebuliser pressure 35 psi. The mass scan range was set as m/z 100–1500.

### Other PGP Activities Determination

Isolate screening for ACC deaminase was determined by ACC dworkin-foster minimal salt (ADF) agar (Honma and Shimomura, 1978), in which 3 mM ACC instead of (NH_4_)_2_SO_4_ as nitrogen source. The inoculated ADF agar was cultured at 28°C for 7 days, and the observed growth was transferred to the second generation. Positive isolate at the fifth generation indicates the ACC deaminase activity. The quantitative assay of ACC deaminase was determined by the amount of α-ketobutyrate (α-KB) production as described as previous (El-Tarabily, 2008; Qin et al., 2014). Phosphate (P-) solubilization activity was determined by using pikovskayas agar (Sigma-Aldrich). The isolate was inoculated onto the pikovskayas agar, cultured at 28°C for 5 days. After incubation, phosphate-solubilizing isolate would form a clear halo zone around the bacterial colony (Qin et al., 2014). Siderophore production was examined by using chrome azurol S (CAS) agar (Schwyn and Neilands, 1987). Isolate was inoculated onto CAS agar, cultured at 28°C for 2 days, and the positive strain was indicated by an orange halo around the bacterial colony.

### Statistical Analysis

Unless otherwise indicated, the experiments of this study were performed in triplicate, and the average value was calculated for the quantitative determinations. GraphPad Prism software was used for data analysis, and data are presented as means ± SD. The difference of bacterial communities between tea cultivars of Zijuan and Yunkang-10 was assessed by PERMANOVA (McArdle and Anderson, 2001), and *p*-values < 0.05 were considered statistically significant.

## Results

### The Catechin and Anthocyan Contents in Zijuan and Yunkang-10

**FIGURE 1:**
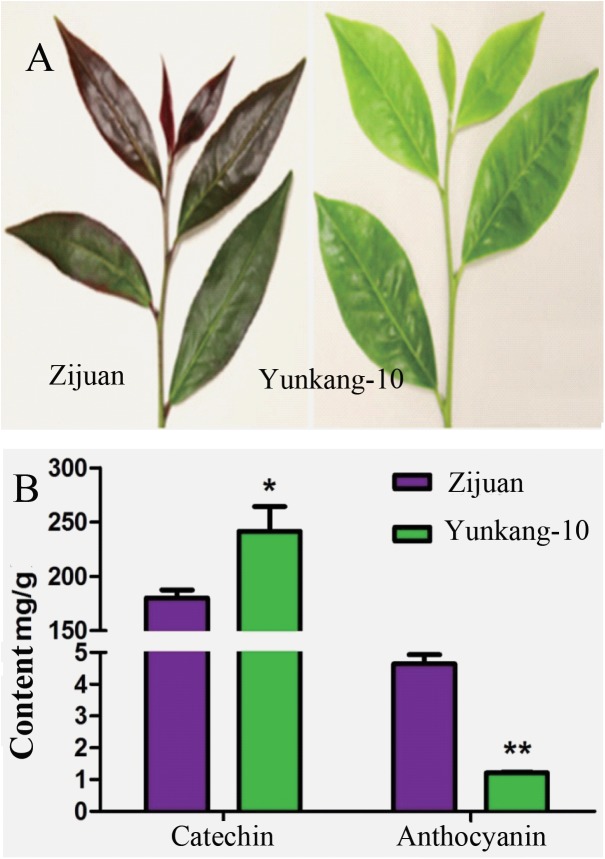
The contents of total catechins and anthocyanins in fresh leaf of Zijuan and Yunkang-10 (^∗^*p* < 0.05, ^∗∗^*p* < 0.01). **(A)** The leaf photographs of Zijuan and Yunkang-10. **(B)** The total contents of catechins and anthocyanins.

The secondary metabolites determination result showed that the catechins content in Zijuan was 180.3 mg/g fresh leaf, and in Yunkang-10 was 241.6 mg/g fresh leaf. In contrast, the anthocyanins content in Zijuan was 4.7 mg/g comparing to 1.2 mg/g in Yunkang-10 fresh leaf, and the high anthocyanins content in cultivar Zijuan is obviously indicated by purple color of the fresh leaf (**Figures 1A,B**). The analysis results showed that the catechins in Zijuan were significantly lower than that of Yunkang-10, while anthocyanins in Zijuan were about 3 ∼ 4 times higher than those of Yunkang-10. The catechins and anthocyans determined in this study were substantial consistent to the previous (Jiang L. et al., 2013), and these secondary metabolites difference might affect microbial colonization (Maximilien et al., 1998).

### The Endophytic Bacteria Isolation Results From Tea Plants

**FIGURE 2:**
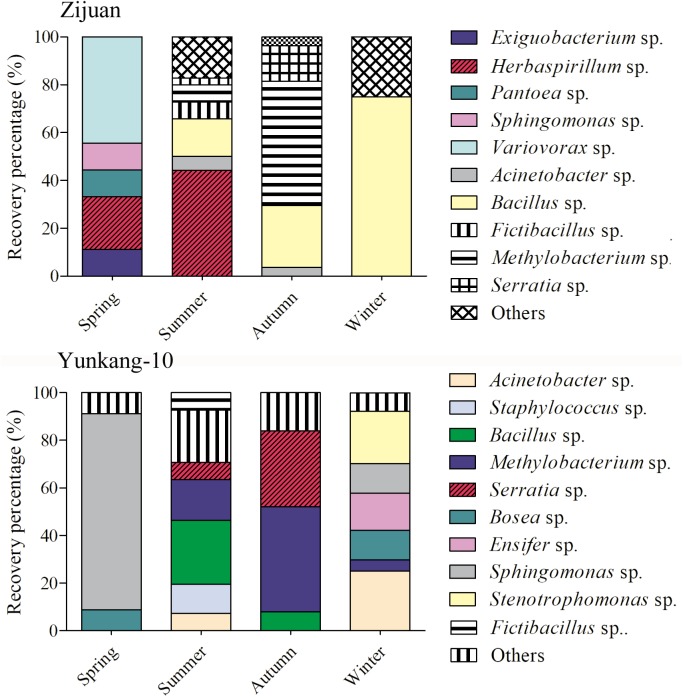
The community dynamics and comparisons for the cultivable endophytic bacteria from Zijuan and Yunkang-10 in four seasons.

The cultivable endophytic bacterial communities from Zijuan and Yunkang-10 were quite different, and the bacterial community in each tea cultivar was always changing from one season to another. For the Zijuan results, a total of nine isolates were obtained in spring, and the dominant groups were *Variovorax* spp. (44.4%) and *Herbaspirillum* spp. (22.2%), the other groups were only obtained one isolate for each genus; 70 isolates were obtained in summer, the dominant groups including *Herbaspirillum* spp. (44.29%) and *Bacillus* spp. (15.7%), and the other groups were less than 10% for each genus; 27 isolates were obtained in autumn, and the dominant groups were *Methylobacterium* spp. (51.9%), *Bacillus* spp. (25.9%) and *Serratia* spp. (14.8%), and the other groups were less than 4.0% for each genus; 4 isolates were obtained in winter, the 3 isolates were *Bacillus* spp. and the other was *Brevundimonas* sp. (**Figure 2** and **Supplementary Table S1**). For the Yunkang-10 results, a total of 34 isolates were obtained in spring, and the predominant group was *Sphingomonas* spp. (82.4%), and the other groups were less than 10% for each genus; 41 isolates were obtained in summer, the dominant groups were *Bacillus* spp. (26.8%), *Methylobacterium* spp. (17.1%), *Staphylococcus* spp. (12.2%), and the other groups were less than 10% for each genus; 25 isolates were obtained in autumn, the dominant groups were *Methylobacterium* spp. (44.9%), *Serratia* spp. (32%), and the other groups were less than 10% for each genus; 64 isolates were obtained in winter, the dominant groups were *Acinetobacter* spp. (25%), *Stenotrophomonas* spp. (21.9%), *Ensifer* spp. (15.6%), *Bosea* spp. (12.5%), *Sphingomonas* spp. (12.5%), and the other groups were less than 5% for each genus (**Figure 2** and **Supplementary Table S1**). As indicated above, *Herbaspirillum* spp. were dominant group in Zijuan on spring and summer; *Methylobacterium* spp. were dominant group in Zijuan on autumn, and in Yunkang-10 on summer and autumn; *Bacillus* sp. were dominant group in Zijuan on summer, autumn and winter, and in Yunkang-10 on summer. Hence, the groups of *Herbaspirillum* spp., *Methylobacterium* spp., and *Bacillus* spp. are the most important endophytic bacteria for tea cultivars of Zijuan and Yunkang-10.

Phylogenetic analysis showed that 110 isolates from Zijuan were belonged to three phylums, while the 164 isolates from Yunkang-10 were belonged to two phylums. Specifically, bacterial groups from Zijuan (**Supplementary Figure S1**) included *Proteobacteria* (nine genera: *Sphingomonas, Brevundimonas, Methylobacterium, Variovorax, Herbaspirillum, Ralstonia, Pantoea, Acinetobacter, Serratia*), *Firmicutes* (eight genera, *Exiguobacterium, Bacillus, Fictibacillus, Bhargavaea, Lysinibacillus, Sporosarcina, Staphylococcus, Oceanobacillus*) and *Bacteroidetes* (one genus, *Myroides*). Comparatively, bacterial groups from Yunkang-10 (**Supplementary Figure S2**) were *Proteobacteria* (16 genera: *Sphingomonas, Bosea, Brevundimonas, Methylobacterium, Sphingobium, Bradyrhizobium, Ensifer, Variovorax, Aquincola, Burkholderia, Massilia, Luteibacter, Serratia, Acinetobacter, Pseudomonas, Stenotrophomonas*) and *Firmicutes* (6 genera, *Bacillus, Fictibacillus, Sporosarcina, Staphylococcus, Oceanobacillus, Paenibacillus*). The mutual bacterial genera of the two cultivars were *Sphingomonas, Variovorax, Acinetobacter, Bacillus, Fictibacillus, Brevundimonas, Methylobacterium, Serratia, Sporosarcina, Staphylococcus, Oceanobacillus*, and *Brevundimonas*. The genera of *Exiguobacterium, Herbaspirillum, Pantoea, Bhargavaea, Lysinibacillus, Myroides*, and *Ralstonia* were exclusively obtained from Zijuan, while the genera of *Aquincola, Bosea, Luteibacter, Sphingobium, Burkholderia, Bradyrhizobium, Pseudomonas, Ensifer, Massilia, Paenibacillus*, and *Stenotrophomonas* were only obtained from Yunkang-10. However, no statistical significant difference was observed (*p* = 0.1) for the bacterial communities between Zijuan and Yunkang-10 determined by perMANOVA tests.

### The Bacterial Diversity of the Two Tea Cultivars

The diversity index of *H*′ expresses the relative complexity of the community structure. The higher diversity index of *H*′ indicates the more abundant species, whereas the *D* index expresses dominance or relative concentration of the importance values to few species (Shannon, 1948; Simpson, 1949; Whittaker, 1972). The evenness index of *J* reflects the uniformity of individual species, and the richness index of *E* strongly depends on the species number which can reflect the species distribution (Menhinick, 1964; Pielou, 1969; Peet, 1974). Due to the reduced number of species isolated in this study (richness equal to one species in some samples), the statistical tests for the diversity analysis could not be properly carried out, and diversity indexes were calculated for the overall treatments and not for single sample. Diversity index (*H*′), evenness index (*J*), dominance index (*D*) and richness index (*E*) of the endophytic bacteria from tea cultivars Zijuan and Yunkang-10 were calculated and compared as follow (**Supplementary Figure S3**). The analysis results showed that the endophytic bacterial diversity indexes of *H*′, *D*, and *E* from Zijuan in summer were higher than those in autumn and winter, but the evenness index of *J* showed non-obviously difference. The bacterial diversity indexs of *H*′, *J, D*, and *E* from Yunkang-10 in summer and winter were higher than those in autumn and spring, and the diversity indexes of spring were the lowest among four seasons. When compared the two cultivars, the endophytic bacterial diversity indexes of *H*′, *J*, and *D* in spring from Zijuan were higher than those of Yunkang-10, and the richness index of *E* showed no obvious difference from each other. Other than the richness index *E* in summer, the diversity indexes of *H*′, *J, D*, and *E* showed consistently higher values in Yunkang-10 than those of Zijuan in summer, autumn and winter. The diversity results indicated that endophytic bacteria of Zijuan were most abundant in summer, but endophytic bacteria of Yunkang-10 were most abundant in winter; the endophytic bacterial diversities in Yunkang-10 were higher than those of the closest tea cultivar Zijuan in summer, autumn and winter.

### PGP Activities of the Endophytic Isolates

*Herbaspirillum* spp. were the dominant group of Zijuan in spring and summer, *Methylobacterium* spp. were dominant group in Zijuan on autumn, and in Yunkang-10 on summer and autumn, *Bradyrhizobium* spp. were recognized nitrogen-fixing bacterial genus. The isolates of *Herbaspirillum, Methylobacterium* and *Bradyrhizobium* were randomly selected and examined for the PGP activities, and the preliminary screening results were summarized in **Table 1**. The screening results showed that 11 selected *Herbaspirillum* isolates showed PGP activities, but only a few *Methylobacterium* isolates displayed PGP activities. Eight *Herbaspirillum* isolates (other than ZXN223, ZXN121, and ZXN122) displayed five tested PGP activities of nitrogen-fixation, P-solubilization, siderophore, IAA production, and ACC deaminase. The *Bradyrhizobium* isolates showed PGP activities of nitrogen-fixation, siderophore and IAA production (or ACC deaminase), but neither of them possessed five PGP activities. IAA biosynthesis was only observed for three *Methylobacterium* isolates, while 10 of 11 *Herbaspirillum* isolates were positive for IAA production. In the phosphate solubilization screening, only *Herbaspirillum* isolates showed appearance of well-developed clearing zones after 5 days incubation, and no isolates from other genera showed the p-solubilization activity.

**Table 1:** The plant-growth promoting (PGP) activity screening on endophytic bacteria from Zijuan and Yunkang-10.

Isolates	Nitrogen fixation	P-solubilization	Siderophore	IAA production	ACC deaminase
***Herbaspirillum* sp.**					
ZXN223	-	+	+	-	+
ZXN4311	+	+	+	+	+
ZXN111	+	+	+	+	+
ZXT112	+	+	+	+	+
ZXT113	+	+	+	+	+
ZXT114	+	+	+	+	+
ZXT117	+	+	+	+	+
ZXN121	-	+	+	+	+
ZXL111	+	+	+	+	+
ZXL112	+	+	+	+	+
ZXN122	-	+	+	+	+
***Methylobacterium* sp.**					
YFY141	+	-	-	+	+
YFY142	-	-	-	-	-
YFY242	+	-	-	+	+
YXT237	-	-	-	-	-
YXN332	-	-	-	+	-
YXT231	-	-	-	-	-
YXT234	-	-	-	-	-
YXT235	-	-	-	-	-
YXN311	-	-	-	-	+
***Bradyrhizobium* sp.**					
YKFG8152	+	-	+	+	-
YKFT8112	+	-	+	-	+


**FIGURE 3:**
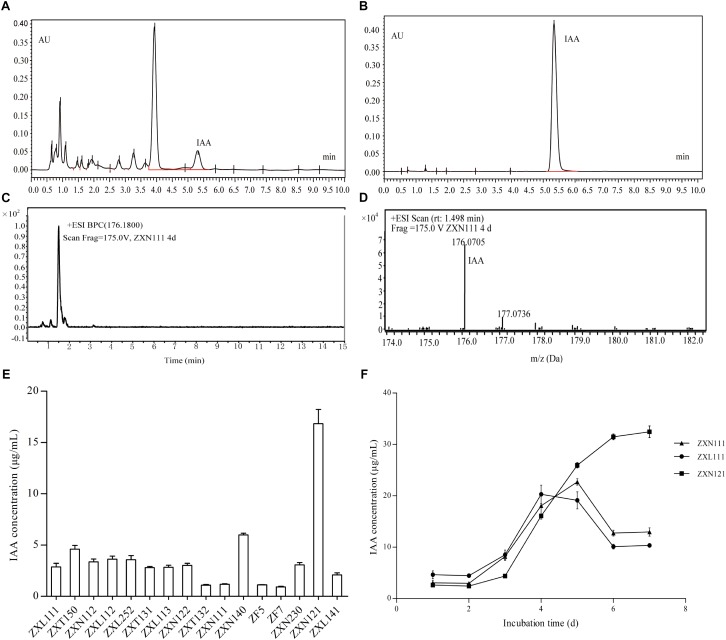
The IAA screening results and accurate determinations on *Herbaspirillum* isolates from Zijuan. **(A)** The IAA extract from isolate ZXN111 by UPLC. **(B)** The IAA standard by UPLC. **(C)** Total ion current chromatogram of IAA extract in UPLC-MS. **(D)** The mass spectra of IAA extract in UPLC-MS. **(E)** The screening results of IAA biosynthesis on endophytic bacteria. **(F)** The IAA biosynthesis dynamics of the three randomly selected *Herbaspirillum* isolates.

On the basis of PGP activity screening, three *Herbaspirillum* isolates were further evaluated for IAA production dynamics by UPLC and UPLC-MS. The results showed that IAA produced from *Herbaspirillum* isolates could be quantified by UPLC using the IAA standards, and identified by UPLC-MS using the distinctive m/z value of 176.07 Da (**Figures 3A–D**). As the quantified results, 16 *Herbaspirillum* isolates all produced IAA during the 4-days incubation, but the IAA biosynthesis yields were quite different among these isolates (**Figure 3E**). The production dynamics of the three selected *Herbaspirillum* isolates showed that the yield of isolate ZXN121 was much higher than those of isolates ZXN111 and ZXL111 after 5-days incubation. The maximal IAA concentration (∼20 μg/mL) in ZXN111 or ZXL111 culture supernatant was observed at 5th day, and then the IAA content gradually decreased. While, the IAA content in ZXN121 culture supernatant was steady increased (>30 μg/mL) during 7 days incubation (**Figure 3F**). However, the most efficient *Methylobacterium* and *Bradyrhizobium* isolates for IAA biosynthesis were *Methylobacterium* YFY141, *Methylobacterium* YFY242, and *Methylobacterium* YXN332, but the maximal yield of the three isolates during 7 days was lower than 3.0 μg/mL (data not shown).

**FIGURE 4:**
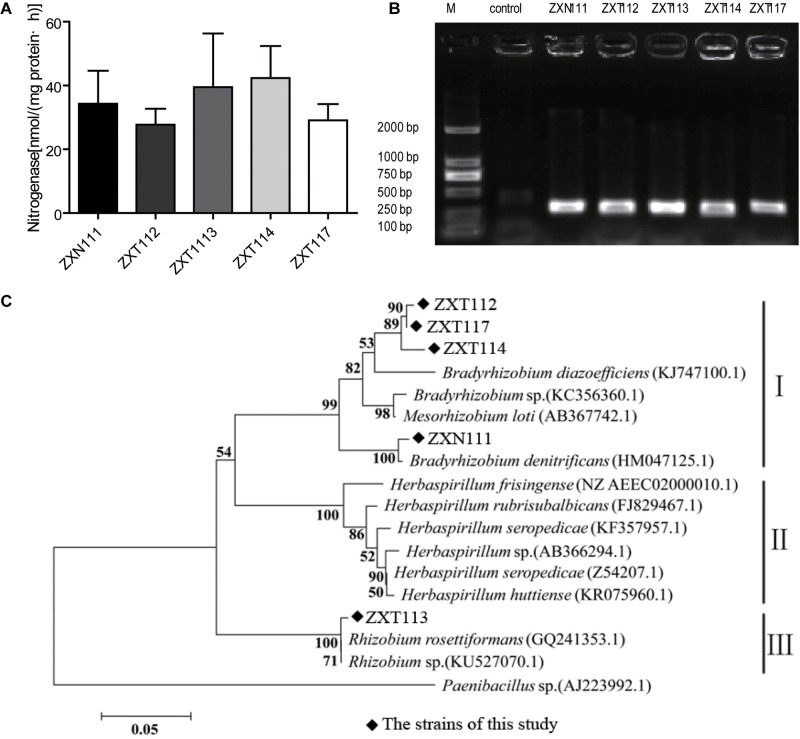
The nitrogen-fixing determination results for the *Herbaspirillum* isolates. **(A)** The result of nitrogenase activity. **(B)** The PCR amplification of *nifH* gene. **(C)** Phylogenetic analysis of the *nifH* gene from *Herbaspirillum* isolates.

The nitrogenase activities of five positive nitrogen-fixing isolates determined by acetylene reduction assay were 34.3, 38.0, 28.5, 42.3, and 29.1 nmol C_2_H_4_/mg⋅protein⋅h (**Figure 4A**), respectively. The nitrogenase activities of this study were higher than *Azotobacter* sp. strain ACCC10006 (19.2 nmol C_2_H_4_/mg⋅protein⋅h), but consistent to *Rhizobium* sp. (30–60 nmol C_2_H_4_/mg⋅protein⋅h; Gibson et al., 1976; Vladimir and Dobereiner, 1988). The PCR products indicated that the five selected nitrogen-fixing isolates contained an amplifiable expected band (∼360 bp) on agarose gel electrophoresis (**Figure 4B**). The similarities of gene *nifH* from the five isolates were 94 ∼ 99% compared with GenBank database. Twelve *nifH* gene sequences with the highest homology to the five *Herbaspirillum* isolates were selected, and the phylogenetic analysis was performed. The *nifH* gene sequences of the five *Herbaspirillum* isolates were clustered to genera of *Rhizobium* and *Bradyrhizobium*, but were clearly separated from other *Herbaspirillum* spp. and *Paenibacillus* sp. obtained from GenBank database. Specifically, the *nifH* gene sequences of ZXT112, ZXT114, ZXT117, and ZXN111 were closely to genus *Bradyrhizobium*, and the *nifH* gene sequence of ZXT113 showed the highest similarity to genus *Rhizobium*. Phylogenetic analysis indicated that the *nifH* gene of *Herbaspirillum* spp. from Zijuan showed the closest relations to *Bradyrhizobia* sp. and *Rhizobia* sp., but far from the type species of *Herbaspirillum seropedicae* (**Figure 4C**).

## Discussion

Tea cultivars of Zijuan and Yunkang-10 are origin from Yunnan Province of China, and two cultivars belong to the same taxonomic species of *C. sinensis* var. *assamic*. The two closest cultivars are the major raw materials for Pu’er tea (dark tea) production. The natural characters and planting environments of the two cultivars are similar, and the leaf anthocyanins content was the major difference (Jiang L. et al., 2013). As indicated in the introduction, tea plants are rich in tea polyphenols, anthocyanins and theanine, and the secondary metabolites have intensive antimicrobial activity to environmental microorganisms (Jiang L. et al., 2013; Jiang X. et al., 2013). Thus, endophytic bacteria of tea plant are relatively scarce, and the endophytes isolation from tea plants is a hard work for microbiologists. This study had tried best to isolate more endophytic bacteria by using different media at different seasons, but only 110 isolates were obtained from Zijuan and 164 isolates were obtained from Yunkang-10 in total. Due to the reduced isolates from two tea cultivars, no significant difference (*p* > 0.05) was observed for the bacterial communities between Zijuan and Yunkang-10 by perMANOVA test. However, the cultivable endophytic bacteria communities of the two cultivars were substantially different. For example, a total of 12 mutual bacteria genera were obtained from the two cultivars, but 18 genera were exclusive for one of the cultivars (i.e., 7 obtained from Zijuan and 11 obtained from Yunkang-10). As the isolation results indicated, *Herbaspirillum* spp. were the dominant and the most important PGP group of tea cultivar Zijuan in spring and summer, but the *Herbaspirillum* sp. was unexpectedly not observed from Yunkang-10 at any season. Similar to *Herbaspirillum* isolates, other dominant groups of the two cultivars were also different. As the results chapter and previous studies indicated (Jiang L. et al., 2013), the difference of the secondary metabolites (especially the of anthocyanins content) in the two cultivars might be the intrinsic factor for the difference of endophytic community (Maximilien et al., 1998). Furthermore, the bacterial community in each tea cultivar was always changing from one season to another. This result was similar to previous microbial community studies on other plant endophytes and environmental samples, and the bacterial communities were influenced by temperature and climate changes, nutrition acquisition, and the activities of other living organisms in different seasons (Bulgari et al., 2014; Li et al., 2014; Resende et al., 2016).

As the same plant species, tea cultivar Yunkang-10 grows better than Zijuan (data not shown) at the same transplanted nursery with equal water and fertilizer management. Conversely, the microbial communities between the two cultivars were quite different, and the PGP endophytes of *Herbaspirillum* spp. in Zijuan of the spring and summer were not observed from Yunkang-10. Isolates of *Herbaspirillum* spp. were considered as the most active PGP endophytes among the evaluated isolates from the two cultivars. Although isolates of *Herbaspirillum* spp. were recognized as the dominant endophytic group of Zijuan in spring and summer, they were not observed from Zijuan at autumn and winter. Meanwhile, isolates of *Herbaspirillum* spp. were not obtained from Yunkang-10 at any season of this study. As we known, spring and summer are the most important seasons for tea plants growth and tea-picking, and nitrogen is in great demand in the two seasons; the dominant group of *Herbaspirillum* spp. (PGP endophytes) in the two seasons should be closest related to plant growth regulations of Zijuan. However, the growth of transplanted Yunkang-10 was obviously better that of Zijuan at the same planting conditions. Why the tea plant growths were not consistent to the PGP endophytes investigated results here? Previous studies indicated that the PGP endophytes of *Klebsiella* sp., *Methylobacterium* sp. assisted the host plants (*Psammochloa villosa, J. curcas* L.) growing on barren dune stubbornly (Chen et al., 2013; Madhaiyan et al., 2013, 2014, 2015). The study of Qin et al. (2014) found that the endophytic isolates with PGP activity of ACC deaminase, IAA synthesis or nitrogen-fixation enabled the hostplant *L. sinense* (Girard) Kuntze grow better under salt stress (Qin et al., 2014). Tea cultivars of Zijuan and Yunkang-10 (*C. sinensis* var. *assamic*) were transplanted from Yunnan Province of China, and the plateau-climate (∼2000 M of elevation) was the origin growth environment and considered more suitable for the plant growth. Compare to Yunnan province, the growth of Zijuan was weak in Anhui province of China, but the growth of transplanted Yunkang-10 was similar as the origin area. The transplanted environments at Anhui province may not appropriate for Zijuan, and the active PGP endophytes assisted the host plant grow at the flat areas, but these endophytes were not obligatory for Yunkang-10.

The nitrogen-fixation results by phenotype and genotype indicated that the majority isolates of *Herbaspirillum* sp. and *Bradyrhizobium* sp., and a few *Methylobacterium* sp. showed the nitrogenase activities; however, the *nifH* gene of *Herbaspirillum* spp. from Zijuan showed the closest relations to *Bradyrhizobia* sp. and *Rhizobia* sp., but not clustered to the typical nitrogen-fixation *Herbaspirillum* species (*Herbaspirillum seropedicae*) of gramineous plants. The *nifH* gene from *Herbaspirillum* spp. of this study might originate from *Bradyrhizobia* sp. or *Rhizobia* sp. (**Figure 4C**) by horizontal gene transfer (Yap et al., 1999), but this presume and the specific nitrogen-fixation mechanism still need to be investigated further. Anyway, the special group of *Herbaspirillum* spp. from Zijuan cultivar is the important PGP endophytes and assists the host plants growing on unfavorite environments in Anhui Province.

## Author Contributions

XY, ZW, YM, LW, and SP performed the experiments. XW and QX analyzed the data and wrote the paper. YZ and CW designed the experiments and reviewed the manuscript. All authors read and approved the final manuscript.

## Conflict of Interest Statement

The authors declare that the research was conducted in the absence of any commercial or financial relationships that could be construed as a potential conflict of interest.

## Footnotes

1 www.ncbi.nlm.nih.gov

## Supplementary Material

FIGURE S1 Neighbor-joining phylogenetic tree based on 16S rRNA gene showing taxonomic results of endophytic bacteria obtained from Zijuan in different seasons. Percentages at the nodes indicate levels of bootstrap support based on neighbor-joining analyses of 1000 resampled datasets.

FIGURE S2 Neighbor-joining phylogenetic tree based on 16S rRNA gene showing taxonomic results of endophytic bacteria obtained from Yunkang-10 in different seasons. Percentages at the nodes indicate levels of bootstrap support based on neighbor-joining analyses of 1000 resampled datasets.

FIGURE S3 The diversity comparisons of endophytic bacteria obtained from tea cultivars of Zijuan and Yunkang-10 in different seasons.

TABLE S1 The endophytic bacteria list obtained from Zijuan and Yunkang-10 in four seasons.
