# Supplementary figures and images for: Isolation, Diversity, and Growth-Promoting Activities of Endophytic Bacteria From Tea Cultivars of Zijuan and Yunkang-10
Source: Front Microbiol. 2018 Aug 21;9:1848. doi: 10.3389/fmicb.2018.01848 (PMC6111150; doi:10.3389/fmicb.2018.01848)

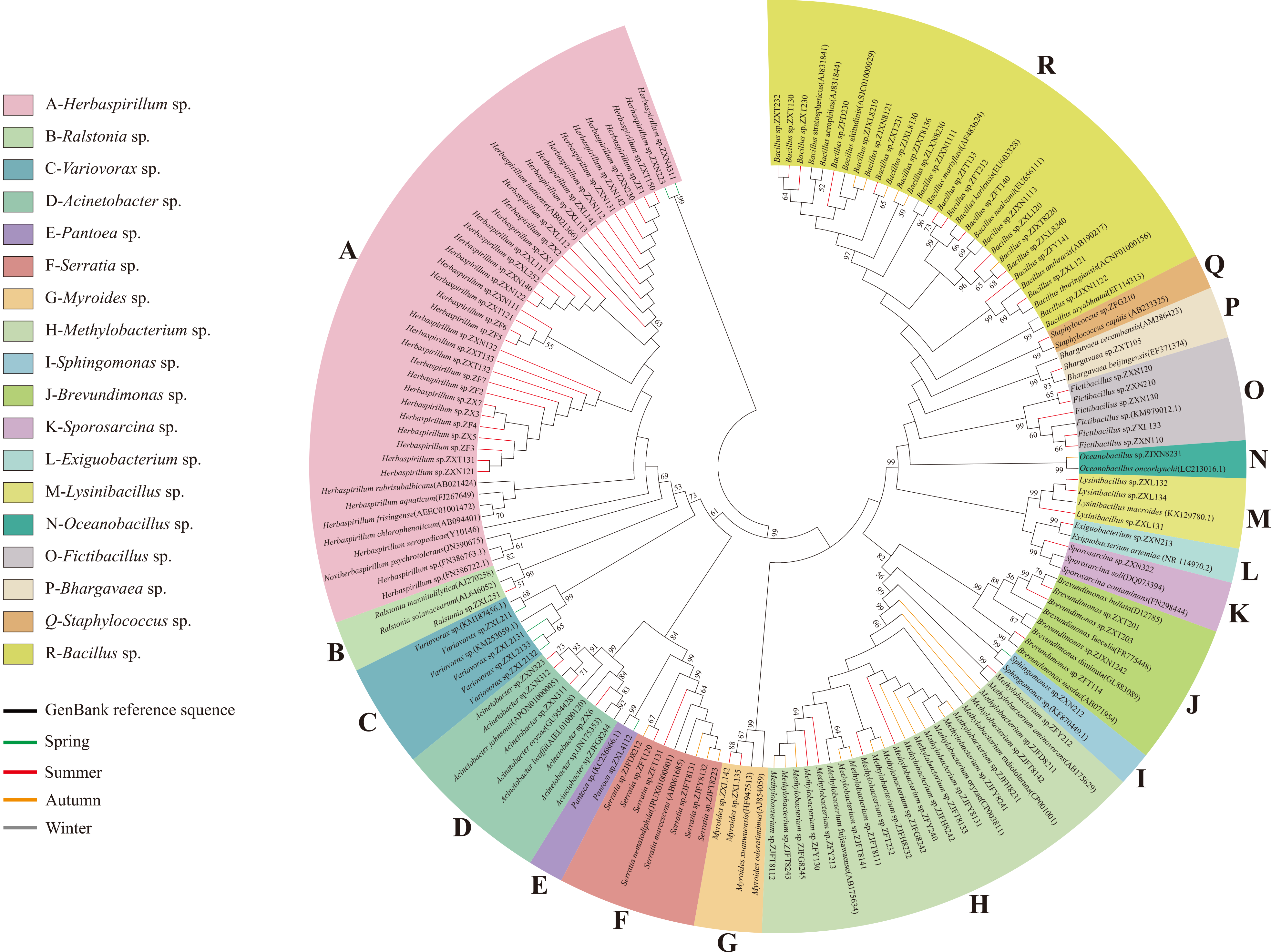

Supplement: FIGURE S1 — Neighbor-joining phylogenetic tree based on 16S rRNA gene showing taxonomic results of endophytic bacteria obtained from Zijuan in different seasons. Percentages at the nodes indicate levels of bootstrap support based on neighbor-joining analyses of 1000 resampled datasets. [file Image_1.TIF]

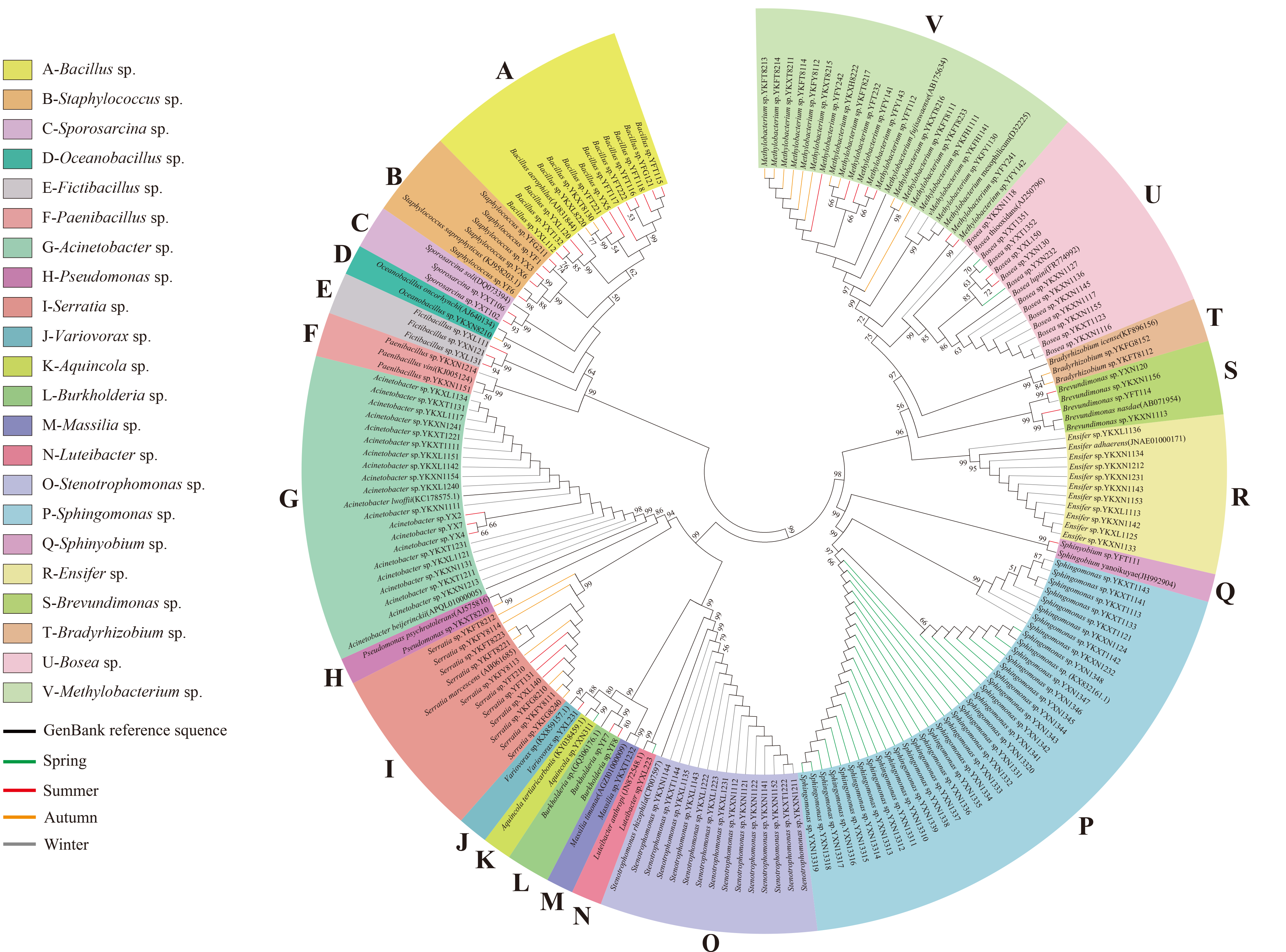

Supplement: FIGURE S2 — Neighbor-joining phylogenetic tree based on 16S rRNA gene showing taxonomic results of endophytic bacteria obtained from Yunkang-10 in different seasons. Percentages at the nodes indicate levels of bootstrap support based on neighbor-joining analyses of 1000 resampled datasets. [file Image_2.TIF]

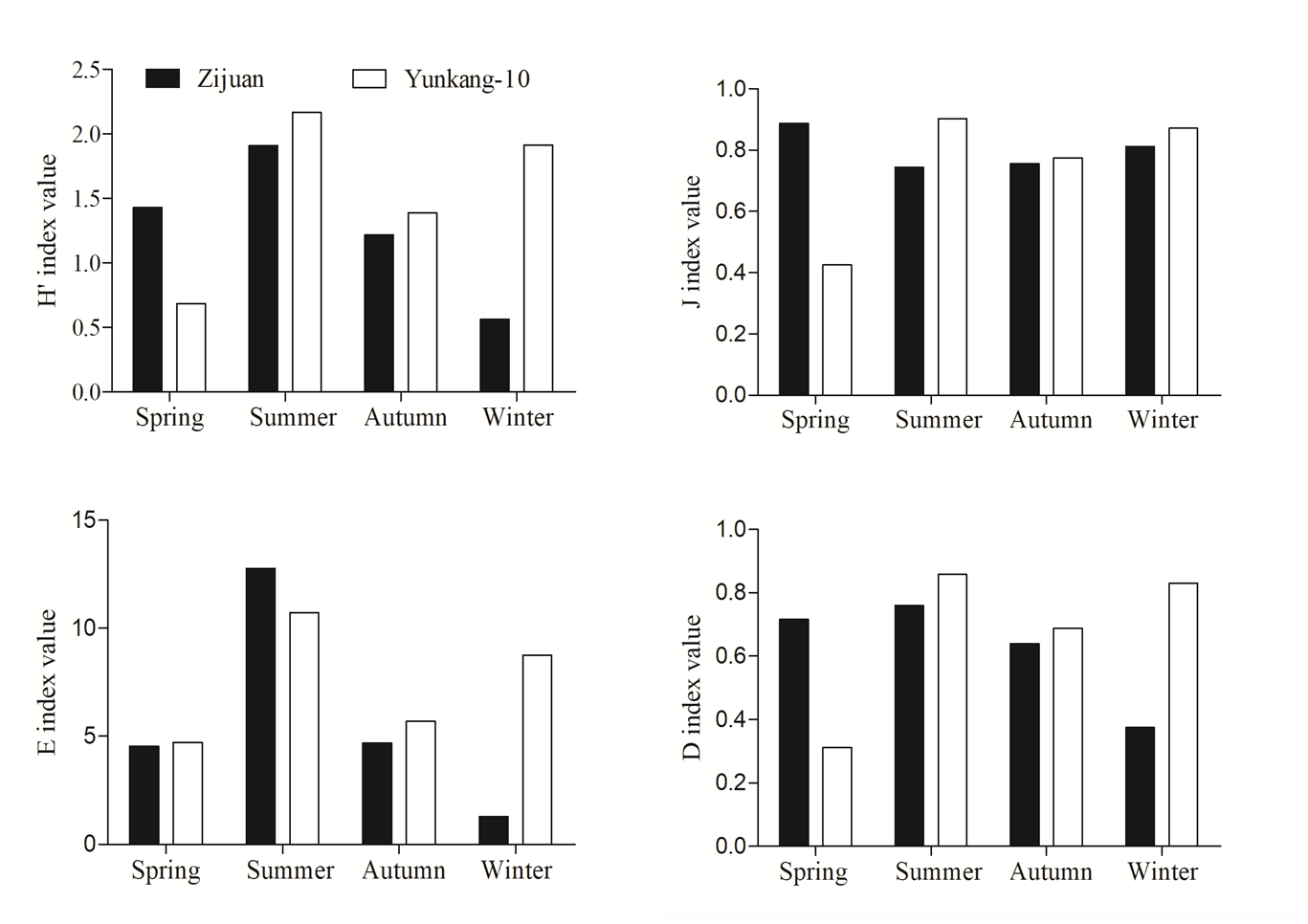

Supplement: FIGURE S3 — The diversity comparisons of endophytic bacteria obtained from tea cultivars of Zijuan and Yunkang-10 in different seasons. [file Image_3.TIF]
