# Supplementary material for: Isolation, Diversity, and Growth-Promoting Activities of Endophytic Bacteria From Tea Cultivars of Zijuan and Yunkang-10
Source: Front Microbiol. 2018 Aug 21;9:1848. doi: 10.3389/fmicb.2018.01848 (PMC6111150; doi:10.3389/fmicb.2018.01848)
Supplement: TABLE S1 — The endophytic bacteria list obtained from Zijuan and Yunkang-10 in four seasons. [file Table_1.DOCX]

|  | Zijuan | | Yunkang-10 | |
| --- | --- | --- | --- | --- |
|  | Species | Number | Sepcies | Number |
| spring | *Sphingomonas* sp. | 1 | *Sphingomonas* sp. | 28 |
|  | *Variovorax* sp. | 4 | *Variovorax* sp. | 1 |
|  | *Exiguobacterium* sp. | 1 | *Aquincola* sp*.* | 1 |
|  | *Herbaspirillum* sp. | 2 | *Bosea sp.* | 3 |
|  | *Pantoea* sp. | 1 | *Luteibacter* sp. | 1 |
|  | **Spring total number** | **9** | **Spring total number** | **34** |
| Summer | *Acinetobacter* sp. | 4 | *Bacillus* sp. | 11 |
|  | *Bacillus* sp. | 11 | *Bosea* sp. | 2 |
|  | *Fictibacillus* sp. | 5 | *Brevundimonas* sp. | 2 |
|  | *Bhargavaea* sp. | 1 | *Fictibacillus* sp. | 3 |
|  | *Brevundimonas* sp. | 3 | *Methylobacterium* sp. | 7 |
|  | *Herbaspirillum* sp. | 31 | *Serratia* sp. | 3 |
|  | *Lysinibacillus* sp. | 3 | *Sphinyobium* sp. | 1 |
|  | *Methylobacterium* sp. | 5 | *Sporosarcina* sp. | 2 |
|  | *Myroides* sp. | 2 | *Staphylococcus* sp. | 5 |
|  | *Ralstonia* sp. | 1 | *Acinetobacter* sp. | 3 |
|  | *Serratia* sp. | 2 | *Burkholderia* sp. | 2 |
|  | *Sporosarcina* sp. | 1 |  |  |
|  | *Staphylococcus* sp. | 1 |  |  |
|  | **Summer total number** | **70** | **Summer total number** | **41** |
| Autumn | *Acinetobacter* sp. | 1 | *Bacillus* sp. | 2 |
|  | *Bacillus* sp. | 7 | *Bradyrhizobium* sp. | 2 |
|  | *Methylobacterium* sp. | 14 | *Methylobacterium* sp. | 11 |
|  | *Oceanobacillus* sp. | 1 | *Oceanobacillus* sp. | 1 |
|  | *Serratia* sp. | 4 | *Pseudomonas* sp. | 1 |
|  |  |  | *Serratia* sp. | 8 |
|  | **Autumn total number** | **27** | **Autumn total number** | **25** |
| Winter | *Bacillus* sp. | 3 | *Acinetobacter* sp. | 16 |
|  | *Brevundimonas* sp. | 1 | *Bosea* sp. | 8 |
|  |  |  | *Brevundimonas* sp. | 2 |
|  |  |  | *Ensifer* sp. | 10 |
|  |  |  | *Massilia* sp. | 1 |
|  |  |  | *Methylobacterium* sp. | 3 |
|  |  |  | *Paenibacillus* sp. | 2 |
|  |  |  | *Sphingomonas* sp*.* | 8 |
|  |  |  | *Stenotrophomonas* sp. | 14 |
|  | **Winter total number** | **4** | **Winter total number** | **64** |

**Supplementary Table 1** The bacterial species list isolated from tea cultivars of Zijuan and Yunkang-10.
